# The natural insect peptide *Neb*-colloostatin induces ovarian atresia and apoptosis in the mealworm *Tenebrio molitor*

**DOI:** 10.1186/1471-213X-14-4

**Published:** 2014-01-30

**Authors:** Elżbieta Czarniewska, Grzegorz Rosiński, Elżbieta Gabała, Mariola Kuczer

**Affiliations:** 1 Department of Animal Physiology & Development, Adam Mickiewicz University, Umultowska 89, 61-614 Poznań, Poland; 2 Department of Cell Biology, Adam Mickiewicz University, Umultowska 89, 61-614 Poznań, Poland; 3 Current address: Research Centre of Quarantine, Invasive & Genetically Modified Organisms, Węgorka 20, 60-318 Poznań, Poland; 4 Faculty of Chemistry, Wrocław University, Joliot-Curie 14, 50-383 Wroclaw, Poland

**Keywords:** Insect ovary, *Neb*-colloostatin, Gonadoinhibitory peptide, Follicle atresia, Apoptosis, Autophagy

## Abstract

**Background:**

The injection of *Neb*-colloostatin into *T. molitor* females causes gonadoinhibitory effects on ovarian development. This peptide inhibits intercellular space formation (patency) in follicular epithelium and results in slowed vitellogenesis, delayed ovulation, reduced number of eggs laid and presumably cell death in the terminal follicles. However, as does the form of cell death in the terminal follicle, the mode of action of *Neb*-colloostatin remains unknown.

**Results:**

We tested *Neb*-colloostatin for a sterilizing effect on females of *Tenebrio molitor*. We report that injection of nanomolar doses of *Neb*-colloostatin induce ovarian follicle atresia in 4-day old females during their first gonadotropic cycle. Light microscope observations revealed morphological changes in the ovary: after *Neb*-colloostatin injection the terminal oocytes are significantly smaller and elicit massive follicle resorption, but the control terminal follicles possess translucent ooplasm in oocytes at different stages of vitellogenesis. A patency is visible in follicular epithelium of the control vitellogenic oocytes, whereas peptide injection inhibits intercellular space formation and, in consequence, inhibits vitellogenesis. Confocal and electron microscope examination showed that peptide injection causes changes in the morphology indicating death of follicular cells. We observed F-actin cytoskeleton disorganization, induction of caspase activity, changes in chromatin organization and autophagic vacuole formation. Moreover, the apical cytoplasm of follicular cells is filled with numerous free ribosomes, probably indicating a higher demand for protein biosynthesis, especially in preparation for autophagic vacuole formation. On the other hand, the process of polyribosomes formation is inhibited, indicating the contributing effect of this hormone.

**Conclusion:**

*Neb*-colloostatin induces atresia in the mealworm ovary. Degeneration of *T. molitor* follicles includes changes in morphology and viability of follicular cells, and oosorption as a consequence of these changes.

## Background

It is commonly acknowledged that insects are capable of producing many eggs in short time intervals. Therefore, oogenesis could be an interesting target for the development of novel strategies for insect population control [1]. The functional unit of the insect ovary is the follicle. It is formed in the anterior region of the ovariole, in the germarium, and is moved posteriorly to the vitellarium developing during oogenesis. Each follicle consists of an oocyte and the surrounding monolayer of somatic follicular cells which constitute the follicular epithelium [2]. Ullmann distinguished nine developmental stages of the oocyte during oogenesis in *T. molitor*; in stages 1–5 oocytes are previtellogenic and in stages 6–9 they are vitellogenic [3]. In early vitellogenic oocyte (stage 6) patency between the follicular cells appears to allow the passage of yolk protein precursors into the oolemma in which they are internalized by endocytosis and deposited as vitellin. As a consequence, a very rapid increase of the oocyte size follows. During midvitellogenesis (stage 7) the follicular epithelium shows maximum patency and yolk deposition, whereas in late vitellogenic oocyte (stage 8) yolk deposition declines, space between follicle cells disappears and follicle cells begin to secrete chorion. The oocyte is mature with fully formed chorion and it is expelled from follicle into lateral oviduct (stage 9) [3].

At present three oostatic peptide hormones have been isolated from insect ovaries: trypsin modulating oostatic factor (*Aea*-TMOF) from *Aedes aegypti*, trypsin-modulating oostatic factor (*Neb*-TMOF) and *Neb*-colloostatin from *Neobellieria bullata*[4]. *Aea*-TMOF discovered by Borovsky et al. [5] in late vitellogenic ovaries of *A. aegypti* females as well as *Neb*-TMOF isolated by Bylemans et al. [6,7] from vitellogenic ovaries of the grey fleshfly *N. bullata*, directly inhibit the biosynthesis of trypsin- and chymotrypsin-like enzymes in the epithelial cells of the insect midgut. This results in the reduction of free amino acids in hemolymph and, in consequence, in the blocking of the biosynthesis of vitellogenin, a protein essential for oocyte growth. *Neb*-TMOF also exerts a strong gonadoinhibitory activity on the ovaries of *Tenebrio molitor* and *Zophobas atratus* beetles [8,9]. Moreover, Hua et al. [10] and De Loof et al. [11,12] have shown that *Neb*-TMOF also inhibits ecdysone biosynthesis by larval ring glands of the fleshflies. *Neb*-colloostatin was discovered during the isolation of *Neb*-TMOF from the ovary of the grey fleshfly. This peptide inhibits yolk uptake by previtellogenic oocytes and, contrary to *Aea*-TMOF and *Neb*-TMOF, it does not inhibit trypsin biosynthesis in the gut or ecdysone biosynthesis by larval ring glands [13]. The gonadoinhibitory properties of *Neb*-colloostatin were confirmed in *T. molitor* ovaries in which it interferes with vitellogenin production by the fat body as well as with vitellogenin uptake by oocytes through modification of patency [9]. Moreover, a novel physiological effect of *Neb*-colloostatin in insects has been recently detected. Injection of *Neb*-colloostatin in physiological concentrations results in significant hemocytotoxicity and a marked increase in apoptotic activity in *T. molitor* haemocytes [14].

Programmed cell death is an active and genetically regulated process eliminating unnecessary or abnormal cells from the organism. Type I programmed cell death, apoptosis, appears to be distinct from autophagic programmed cell death (type II). Apoptotic cell death is mainly characterized by condensation of chromatin at the nuclear membrane, caspase activation, fragmentation of nuclei and cells, and cell fragmentation into apoptotic bodies, whereas organelles are well preserved and autophagocytosis is absent [15-17]. The activation of caspases, the key mediators of this type of cell death, is responsible for the cleavage of cellular target proteins and, in consequence, the observed morphological changes. During autophagic cell death, the autodegradation of cytoplasmatic components takes place, and therefore it is thought that autophagy mainly has a cytoprotective role under stress stimuli [18,19].

Follicular atresia, defined as the degeneration of the follicle, generally occurs during normal oogenesis in many animal species [20,21]. Under physiological conditions it plays a significant role in the maturation process during the normal development of eggs and in the removal of abnormal or damaged oocytes before they reach maturity. In *Drosophila*, Giorgi and Deri [22] found that some egg chambers degenerate almost exclusively between stages 7 and 8 of oogenesis. Other studies of dipteran ovarian follicles confirmed the activation of programmed cell death during mid-oogenesis and late-oogenesis. Therefore apoptotic death of both the nurse cells and the follicle cells in polytrophic ovaries may be observed in either the developmental phase or the degenerative phase of dipteran oogenesis [23-26]. While the nurse cells exhibit the morphological hallmarks of apoptosis, the follicle cells remain remarkably intact and phagocytose the cellular remnants of the nurse cells after they have completed their apoptotic program [23,24,27,28]. However, follicular atresia has been massively induced in response to starvation, malfunction of ecdysone signalling or treatment with chemotherapeutic drugs [23,27-30]. Pathogen infection can also lead to atresia of the ovarian vitellogenic follicles as recently shown in *Rodnius prolixus*[31].

In the present study, we describe *Neb*-colloostatin-induced atresia in the mealworm ovary. Degeneration of *T. molitor* follicles includes changes in morphology and viability of follicular cells, and oosorption as a consequence of these changes.

## Results

### *Neb*-colloostatin injection leads to resorption of vitellogenic ovarian follicles in *T. molitor*

**Figure 1:**
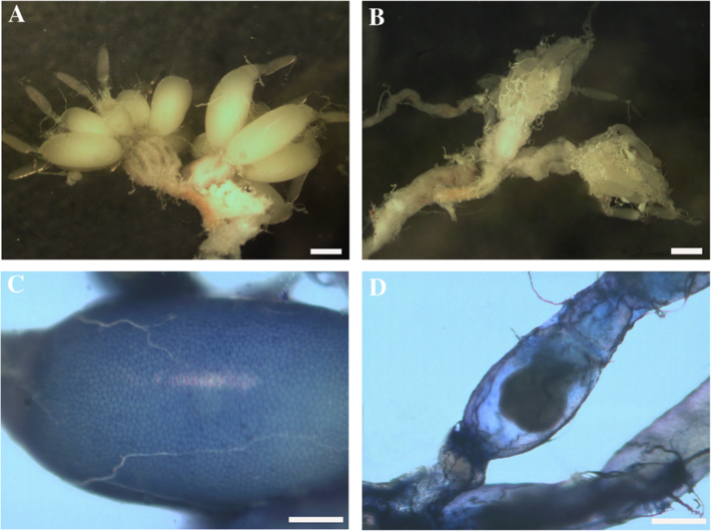
**Light micrographs of ovaries (A-B) and follicles (C-D) of *T. molitor*.** Saline controls **(A, C)**, treated **(B, D)**. Ovary of control female shows large terminal follicle with oocytes full of yolk **(A)**, ovary of treated female has small atretic follicles **(B)**. Terminal follicle of control shows intracellular spaces between follicle cells (patency) stained with 1% Evans blue **(C)**, terminal follicle of treated females shows no Evans blue stain indicating lack of patency **(D)**. Females were injected with saline (control) or 10 nmole *Neb*-colloostatin (treated) on day 3 and assayed on day 4. Scale bars: 1 mm **(A, B)** and 0.5 mm **(C, D)**.

Injection of *Neb*-colloostatin (in doses of 1 or 10 nmole of peptide/female) into the hemocoel of *T. molitor* females during their first reproductive cycle showed that this peptide strongly inhibited ovarian growth and oocyte development (Figure 1). The terminal oocytes of the peptide-injected females were significantly smaller and many follicles showed resorption in contrast to terminal oocytes of control ovaries that were larger and in midvitellogenesis. The control follicles possessed oocytes with translucent ooplasm, whereas follicles from the ovaries of females injected with *Neb*-colloostatin showed oocyte ooplasm alterations under the stereomicroscope (Figure 1). The terminal oocytes are much longer and wider in the control ovary than in that from the peptide injected female (Figure 1A-B) and patency in follicular epithelium is obvious in the control but not in the ovary from the injected female (Figure 1C-D).

### Apoptosis and autophagy are involved in follicle cell death after *Neb*-colloostatin injection

After morphological observations, the entire ovaries were stained in order to detect peptide-induced changes in the organization of the F-actin cytoskeleton and chromatin, and presence of indicators of cell death of the terminal ovarian follicles.

**Figure 2:**
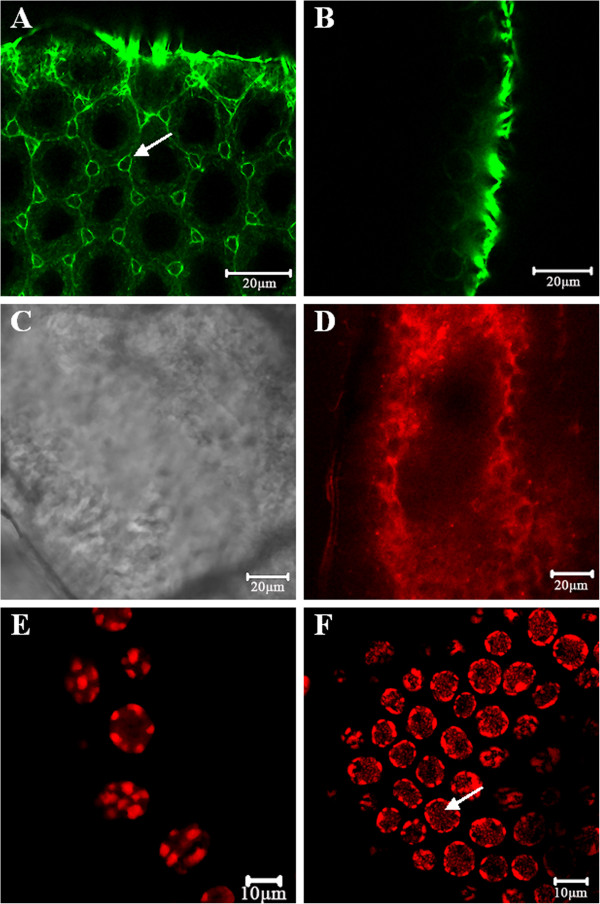
**Confocal micrographs of control (A, C, E) and *Neb*-colloostatin-treated (B, D, E) terminal follicles of *T. molitor*.** In control follicular cells F-actin stained with Oregon Green phalloidin is localized subcortically and maximum patency is visible (arrow) **(A)**. Follicular cells of treated females show no Oregon Green phalloidin stain indicating depolimerization of F-actin **(B)**. In control follicular cells stained with SR-VAD-FMK active caspase is not detected **(C)**. In follicular cells of treated females SR-VAD-FMK stain shows caspase activation **(D)**. In control follicular cells stained with propidium iodide, nuclei have normal pattern of chromatin **(E)**. In follicular cells of treated females stained with propidium iodide, nuclei are shrunk, chromatin organization is changed and DNA fragmentation is visible (arrow) **(F)**. Females were injected with saline (control) or 10 nmole *Neb*-colloostatin (treated) on day 3 and assayed on day 4.

The first marker, Oregon Green 488 phalloidin, delimited changes in the F-actin cytoskeleton. In the control follicular cells, a strong fluorescent signal was localized subcortically (Figure 2A), and was also detected in the oocyte (see Additional file 1). Maximum patency of follicular epithelium was visible. Injection of *Neb*-colloostatin caused failure of F-actin organization in these cells. They lost their regular F-actin staining pattern as a result of depolymerization of F-actin microfilaments. The application of 10 nmole of peptide caused full depolymerization of F-actin, therefore the positive Oregon Green-phalloidin staining signal was not detected (Figure 2B), but a lower dose of the peptide (1 nmole) inhibited intracellular space formation in the follicular epithelium (see Additional file 1).

The sulphorhodamine derivative of valylalanylaspartic acid fluoromethyl ketone (SR-VAD-FMK) staining showed that *Neb*-colloostatin induced the activation of caspases in the ovarioles (Figure 2C-D). In this assay, we observed that the degree of caspase activation depended on the applied hormone dose. One day after injection of *Neb*-colloostatin, at a dose of 1 or 10 nmole per insect, caspase activity was detected in all ovaries of the studied females. Injection of 1 nmol of peptide caused caspase activation only in oocytes (see Additional file 1). Injection of *Neb*-colloostatin at a dose of 10 nmole/female activated caspases in the follicle cells, whereas the oocyte was resorbed (Figure 2D).

Next, we used propidium iodide staining to visualize changes in chromatin organization (Figure 2E-F). The nuclei of follicular cells from ovarian follicles of females injected with *Neb*-colloostatin (at a dose of 10 nmol of peptide) were characterized by highly condensed chromatin redistributed along the nuclear membrane as compared to the control. Moreover, in the nuclei of follicular cells from the peptide-injected females, propidium iodide revealed DNA fragmentation events visible as small, red dots, compared to the control nuclei, the morphology of which looked normal.

**Figure 3:**
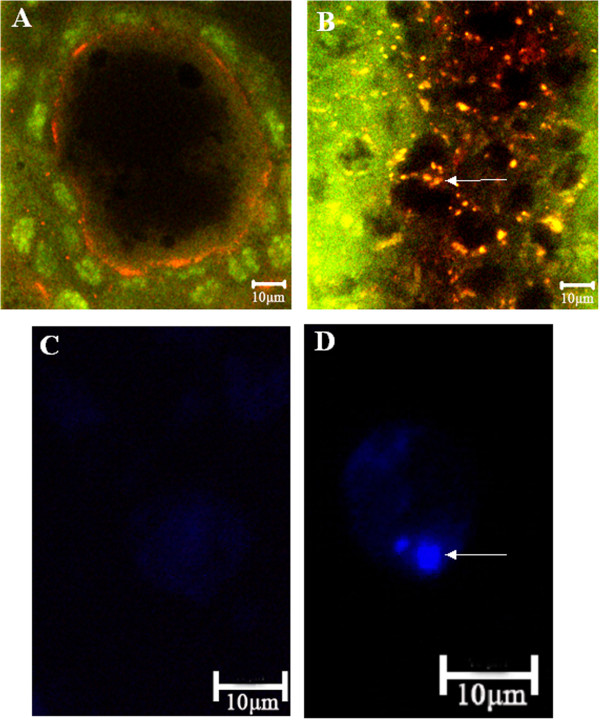
**Confocal and fluorescent micrographs of control (A, C) and treated (B, D) terminal follicles of *T. molitor*.** In control follicular cells stained with orange acridine, lysosomes are not detected and nuclei of follicular cells (green colour) and oocyte (dark colour) are visible **(A)**. In the follicular cells of treated females orange acridine stain shows many lysosomes (yellow colour, arrow) **(B)**. In isolated control follicular cells stained with MDC, autophagic vacuoles are not detected **(C)**. In isolated follicular cell of treated females stained with MDC arrow shows autophagosome (blue colour) **(D)**. Females were injected with saline (control) or 10 nmole *Neb*-colloostatin (treated) on day 3 and assayed on day 4.

The mono-dansyl-cadaverin (MDC) and the orange acridine staining were used for tracing the induction of autophagic vacuoles and detection of lysosomes in order to assess the type of programmed cell death, i.e. type I or type II, caused by the injection of *Neb*-colloostatin (Figure 3). After *Neb*-colloostatin injection at a dose of 10 nmol of peptide per female, fluorescent and confocal microscopy revealed extensive vacuolization of the cytoplasm of follicular cells indicating degeneration of their contents in an autophagy-like process. Orange acridine staining showed the presence of lysosomes in follicular cells of terminal follicles of peptide-injected females (Figure 3B), whereas autophagic vacuoles detected by MDC staining were visible as distinct dot-like blue structures (Figure 3D).

### *Neb*-colloostatin injection induces changes in the ultrastructure of ovarian follicles

In the *T. molitor* ovary, the distinct differences in ultrastructure between the control follicles and the follicles of the *Neb*-colloostatin injected females (in dose of 1 or 10 nmole of *Neb*-colloostatin per female) become visible.

**Figure 4:**
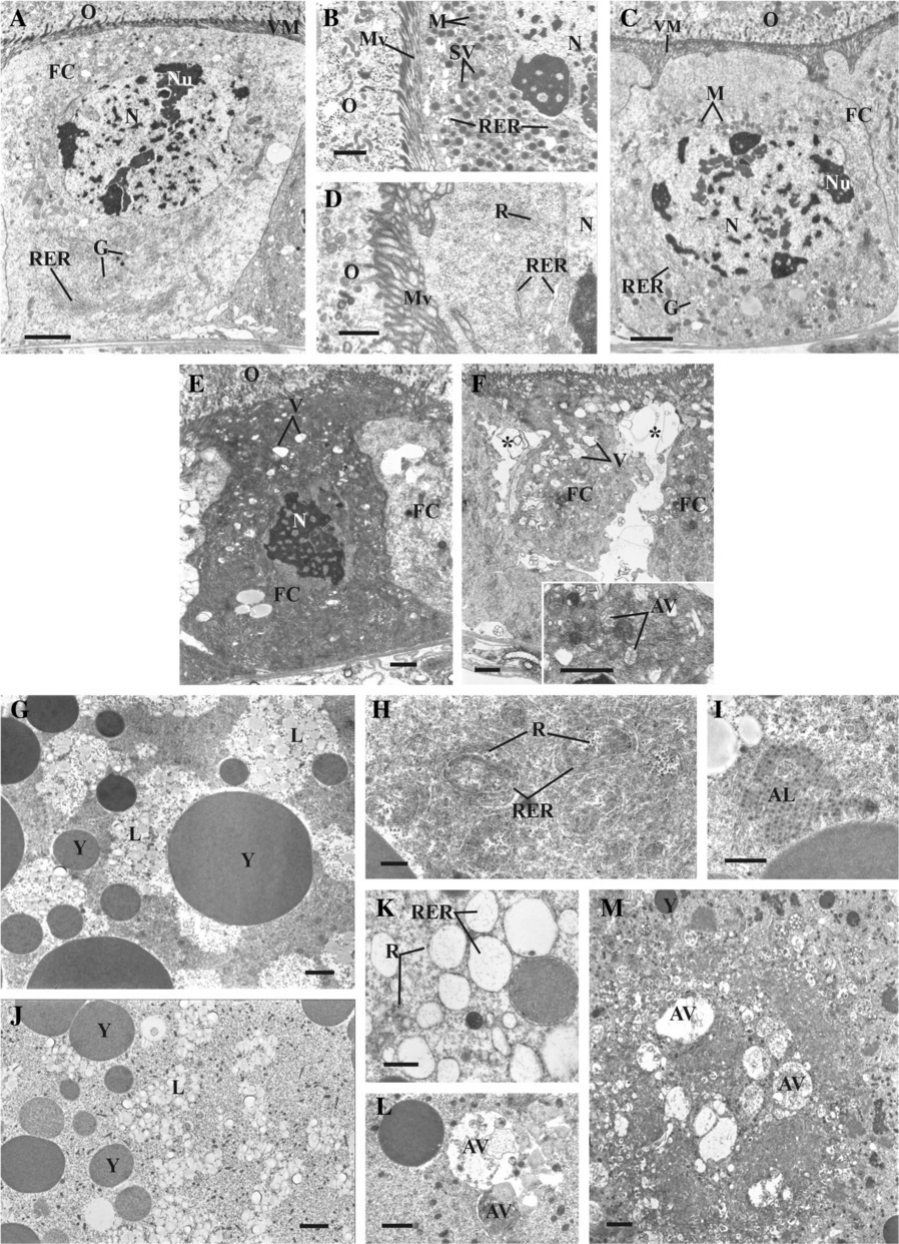
**Ultrastructure of follicular epithelium (A-F) and oocytes at 7th stage of development (G-M) in *T. molitor *ovary.** Follicle cells of control females showing typical pattern **(A, B)**. Low magnification of follicle cell (FC) with well-developed rough endoplasmic reticulum (RER) and Golgi complexes (G) **(A)**. Details of apical part of follicle cell rich in secretory vesicles (SV), mitochondria (M) and rough endoplasmic reticulum (RER) **(B)**. Follicle cells of treated females **(C, D)**. Low magnification of follicle cell (FC) filling with free ribosomes (R) **(C)**. Details of apical part of follicle cell with poorly developed rough endoplasmic reticulum (RER) and Golgi complexes (G) **(D)**. Follicle cells (FC) having numerous vesicles (V) and nucleus (N) with highly condensed chromatin **(E)**. Shrunken follicle cells (FC) forming intercellular spaces (asterisk) **(F)**; inset: autophagic vacuoles (AV) in follicle cell. Oocytes of control females displaying normal pattern **(G-I)**. Cortical ooplasm rich in yolk granules (Y) and lipid droplets (L) **(G)**. Ooplasm with abundance of rough endoplasmic reticulum (RER) and free ribosomes (R) **(H)**. Annulate lamellae (AL) in ooplasm **(I)**. Oocytes of treated females **(J-M)**. Cortical ooplasm with small yolk granules (Y) and lipid droplets (L) **(J)**. Rough endoplasmic reticulum (RER) and ribosomes (R) in ooplasm **(K)**. Cortical ooplasm with autophagic vacuoles (AV) **(L)**. Core of the oocyte containing autophagic vacuoles (AV) **(M)**. M – mitochondria; Mv – microvilli; N - nucleus; Nu – nucleolus; O – oocyte; RER - rough endoplasmic reticulum; V – vacuoles; VM – vitelline membrane. Scale bars: **(A, C, G, J, M)** 2 μm; **(B, E, F, inset, I, L)** 1 μm; **(D, H, K)** 0.5 μm. Females were injected with saline (control) or 10 nmole *Neb*-colloostatin (treated) on day 3 and assayed on day 4.

In the control follicles, the follicular epithelium surrounding the terminal oocyte at the 7th (midvitellogenic) stage of development [3] is composed of the cuboid cells with microvilli directed to the oocyte. The nucleus of the follicle cells contains quite a number of the small clumps of heterochromatin and several vast irregular nucleoli. The follicle cells exhibit characteristic morphology of active secretion. In the basal part of the follicle cells, the cisterns of the rough endoplasmic reticulum (RER) and the Golgi complexes are well-developed (Figure 4A). The apical part is rich in the electron dense secretory vesicles, mitochondria and the dilated cisterns of RER (Figure 4B).

In the follicles of injected females, the follicle cells surrounding the terminal oocyte show no morphological evidence for synthetic activity. A part of the follicle cells is still cube-shaped, although their apical surfaces become more or less wrinkled. The nuclei of these cells usually contain a granular nucleoplasm and a few extensive and irregular nucleoli. Less and smaller, than in the control, heterochromatin clumps are visible. The cisterns of RER are clearly less developed than in the cells of the control follicles (Figure 4A-C). In the apical cytoplasm, there are no secretory granules and a few elements of RER and mitochondria are located in the vicinity of the nucleus (Figure 4C-D). This area is filled with numerous free ribosomes (Figure 4D).

After injection of *Neb*-colloostatin, many follicle cells reveal much more advanced changes. These cells are irregular in shape and their cytoplasm is vacuolated in the varying degrees. In the nuclei of these cells, chromatin becomes highly condensed (Figure 4E). In cytoplasm, the detected vacuoles are electron-transparent (Figure 4E-F) or include the degradated fragments of cytoplasm (Figure 4F inset). The cells shrink and gradually lose their connection with the neighboring follicle cells and the basal lamina (Figure 4F).

In the control follicle, between the terminal oocyte at the 7th stage of development and the follicle cells, is a narrow cleft filled with the electron dense material, forming a vitelline membrane surrounding the oocyte (Figure 4A). The cortical ooplasm in particular contains numerous yolk granules and lipid droplets. (Figure 4G). Free ribosomes, rough endoplasmic reticulum systems (Figure 4H) and stacks of annulate lamellae (Figure 4I) are also abundant in the ooplasm.

In the follicle of females injected with *Neb*-colloostatin, the intercellular cleft between the surface of the follicular epithelium and oocyte is wider than in the control follicle (Figure 4A-C). The cortical region of ooplasm contains fewer and significantly small yolk granules but relatively numerous lipid droplets (Figure 4G-J). The number of free ribosomes is dramatically reduced. The rough endoplasmic reticulum is less developed. It displays vesicles with few ribosomes attached (Figure 4K). Stacks of annulate lamellae are not found in the ooplasm.

In the cortical ooplasm, in between the yolk granules are autophagic vacuoles containing materials in various degrees of degradation (Figure 4L). The core of oocyte is usually devoid of yolk granules and the autophagic vacuoles with a highly degraded content are particularly numerous (Figure 4M).

## Discussion

This study describes the process of atresia in ovarian follicles induced by the gonadoinhibitory peptide *Neb*-colloostatin during the first gonadotropic cycle of *T. molitor* females.

In recent years, several control strategies have been proposed as species-specific and non-polluting methods for reducing or suppressing pest reproduction, especially insect–pathogen interactions or development of the sterile insect technique [31,32]. As previously reported [9], injection of *Neb*-colloostatin and *Neb*-TMOF into *T. molitor* females causes gonadoinhibitory effects on ovarian development, however, the mode of action of these peptides remains unknown. Wasielewski and Rosiński [9] showed that the injection of approximately 2 mmole of *Neb*-coollostatin into females on days 1, 2 and 3 of the first reproductive cycle inhibits patency formation, slowing down vitellogenesis and ovarian development, delaying ovulation and ovarian development, and reducing the number of eggs laid. In *T. molitor*, part of the inhibitory process of the oocytes growth is manifested by qualitative and quantitative changes in proteins sequestered and synthesized by the ovary. The authors showed that the injections of *Neb*-colloostatin reduced by about 60% total protein content in ovaries and induced qualitative changes in ovarian protein patterns. Electrophoretic analysis pointed out that *Neb*-colloostatin caused a loss of two polypeptides and a strong reduction of four another. All these polypeptides were the most abundant in ovaries of the control females. On the other hand, *Neb*-colloostatin injection increased the concentration of two polypeptides, which were found in very small amounts in ovaries of control females. However, *Neb*-colloostatin did not cause a significant change in protein composition of the haemolymph [9]. It seems that *Neb*-colloostatin reduces proteins sequestration by ovaries and the peptide action is more specific to ovaries.

In this paper, we described the morphological alterations caused by *Neb*-colloostatin injections in the oocytes and the follicular epithelium of *T. molitor* egg chambers. Microscopic observation and molecular analysis of the ovarian follicle facilitated a more detailed characterization of the gonadoinhibitory action of this peptide. First, the doses of peptide injected into females applied in this work were much lower than those of Wasielewski and Rosiński [9]. This suggests that the peptide is very potent and could be acting at a physiologically relevant concentration. Second, a very important phase of oocyte growth is the formation of intercellular spaces in the follicular epithelium enabling the transport of nutrients to the growing vitellogenic oocytes [3]. Our findings show that the direct action of *Neb*-colloostatin on follicular cells resulting in F-actin cytoskeleton disorganization, and in consequence inhibition of patency formation, may prompt peptide-induced follicular atresia. Peptide injections strongly inhibited oocyte development. Next, apoptosis was switched on in affected follicles and, in consequence, resorption of oocyte remnants occurred. In the *T. molitor* follicular epithelium, apart from the disorganization of the F-actin cytoskeleton network, other alterations comprised typical features of apoptosis including caspase activation, chromatin condensation and DNA fragmentation. Moreover, evidence for a distinct type of physiological cell death, autophagocytosis, was also observed in oocytes and follicular cells. Incorporation of cellular contents into autophagosomes suggests that these cell components can be released into the insect haemolymph as a result of lysosomal enzyme activity. During autophagy all cellular contents are disintegrated into autophagic vacuoles and destroyed by the cell’s lysosomes [33]. Aguirre et al. [34] showed in *Dipetalogaster maxima* that atresia induced by food deprivation was characterized by loss of the ability of oocytes to uptake vitellogenin, partial vitellin proteolysis and activation of cathepsin D-like peptidases. These authors suggested that cathepsin D-like peptidase is involved in the process of follicle degeneration, probably by promoting early yolk protein degradation, and would also be involved in the oosorption of atretic follicles. Apoptosis can also be detected during extensive autophagocytosis in the course of insect oogenesis. For example, Uchida et al. [25] realized that apoptosis may occur in the epithelial cells of degenerating follicles in the final stages of the atretic process within developing mosquito ovaries. These authors detected caspase and catepsin-like proteinase activation, as well as the presence of fragmented DNA in the epithelial region of the *Culex pipiens pallens* atretic follicles but not in normally developing follicles within the same ovaries. Autophagosomes have also been observed in electron micrographs of degenerating mid-stage follicles from *Drosophila virilis*[35,36]. Valentzas and colleagues [26] suggested that apoptosis and autophagy act synergistically to achieve more efficient elimination of degenerated nurse cells and abnormal eggs chambers. Boolmart et al. [37] suggested that autophagocytosis depends on an intact cytoskeleton. Thus intermediates and microfilaments are considered to be essential for the initial formation of autophagosomes, whereas their subsequent fusion with lysosomes depends on microtubuli [33,37]. In spite of the *Neb*-colloostatin-induced disorganization of the F-actin cytoskeleton network in follicular cells, we observed the occurrence of autophagosomes, however, we cannot explain these data at present. Moreover, electron microscopy of *Neb*-colloostatin-induced changes in the *T. molitor* ovary showed that the apical cytoplasm of the follicular cells is filled with numerous free ribosomes, suggesting that after peptide injection the follicular cells probably have a higher demand on protein biosynthesis, especially in the perspective of increased formation of the autophagic vacuoles process. This result shows that the process of polyribosomes formation can be inhibited, indicating the contributing effect of *Neb*-colloostatin.

## Conclusion

In summary, our results show that in *T. molitor* females, *Neb*-colloostatin injection resulted in atresia of ovarian follicles both the apoptotic and the autophagic mechanism of programmed cell death. The first observable effect of *Neb*-colloostatin-induced atresia is the disappearance of patency in the follicular epithelium. This probably inhibits the active uptake of vitellogenins by the oocyte. Induction of apoptosis and autophagy of ovarian follicles may contribute to more efficient removal of atretic follicles during ovarian follicular regression in insects.

## Methods

### Insects

A stock culture of *T. molitor* was maintained at the Department of Animal Physiology and Development as described previously [38]. Ten females and ten males were mated just after emergence. The control and the experimental groups were kept at the same population density in separate boxes. Studies were carried out on adult females during their first gonadotropic cycle and fifteen ovaries were observed for each treatment.

### Peptide synthesis

*Neb*-colloostatin (SIVPLGLPVPIGPIVVGPR) was synthesized by the classical solid phase method based on the Fmoc-protocol [39] as described previously [8].

The peptide was dissolved in physiological saline for *Tenebrio* (274 mM NaCl, 19 mM KCl, 9 mM CaCl_2_) to yield a stock solution of 1 mM, and it was stored at -30°C. The working dilutions from the stock solution were made in saline.

### Peptide injection and ovary preparation

The 3-day old *T. molitor* female beetles (stage 6 of terminal oocytes) were anaesthetised with CO_2_, washed in distilled water and disinfected with 70% ethanol. *Neb*-colloostatin was injected (2 μl, in a dose of 1 or 10 nmole of peptide per insect) through the ventral membrane between the second and the third abdominal segments towards the head with a Hamilton syringe (Hamilton Co.). The control 3-day old females were injected with the same volume of physiological saline. All solutions were sterilised through a 0.22 μm pore filter membrane (Millipore) and all injections were performed in sterile conditions. Ovaries were dissected 1 day after injection, from CO_2_ anaesthetised females, as described previously [9]. Ovaries were subsequently used for microscopic analysis.

### Morphological and biochemical hallmarks of peptide-induced degeneration of ovarian follicles

#### Ovarian development bioassay

To assess the effect of *Neb*-colloostatin injection on morphology and follicle development, *T. molitor* females were dissected and ovaries were examined with an Olympus SZX 12 (Olympus Optical) stereoscopic microscope. Freshly dissected ovaries were stained with a 1% solution of Evans blue in physiological saline as described by Ullmann [3] for observation of follicular epithelium patency and evaluation of the developmental stage of the oocyte.

#### Detection of F-actin microfilaments

For visualization of F-actin microfilaments, ovaries from control- and peptide-injected females were fixed for 30 min in 4% paraformaldehyde in physiological saline and then permeabilized in 3.7% paraformaldehyde in physiological saline containing 0.1% Triton X-100 for 25 min at room temperature. Next, the ovaries were washed three times with saline and stained with Oregon Green® 488 phalloidin (Invitrogen) for 20 min at room temperature in the dark, according to the manufacturer’s instructions. Thereafter, ovaries were washed twice with physiological saline, mounted using a mounting medium and examined with a Carl Zeiss LSM 510 confocal laser scanning microscope.

#### Active caspase *in situ* assay

Caspase activity was assessed by using a sulphorhodamine derivative of valylalanylaspartic acid fluoromethyl ketone, a potent inhibitor of caspase activity (SR-VAD-FMK; the sulphorhodamine multi-caspases activity kit, AK-115, BIOMOL, PA), in accordance to the method modified by Uchida et al. [25]. Briefly, ovaries from control- and peptide-injected females were rinsed with physiological saline, incubated in reaction medium (1/3 × SR-VAD-FMK) for 2 h at room temperature in the dark, rinsed again with wash buffer overnight at 4°C and finally fixed in 3.7% paraformaldehyde for 10 min. After washing with physiological saline, the prepared ovaries were mounted in antifading mounting medium and studied with a Zeiss LSM 510 confocal laser scanning microscope with filters set for rhodamine (excitation 543 nm and emission 560 nm).

#### Propidium iodide staining

For visualizing chromatin condensation propidium iodide staining was used. Ovaries from control- and peptide-injected females were fixed for 30 min in 4% paraformaldehyde in saline and subsequently permeabilized for 25 min in 4% paraformaldehyde in saline with 0.1% Triton X-100. After three washes in saline for 5 min each, the ovaries were incubated with a solution of RNase A (600 μg/ml) in saline for 1 h at 30°C, washed three times with saline and stained with propidium iodide (700 ng/ml) for 15 min in the dark and at room temperature. Then, the ovaries were washed in saline for 5 min, mounted in antifading mounting medium and viewed using a Carl Zeiss LSM 510 confocal laser scanning microscope.

#### MDC staining for autophagic vacuoles

Autophagic vacuoles were detected by mono-dansyl-cadaverin (MDC) staining according to the method of Biederbick et al. [40]. Ovaries from control- and peptide-injected females were briefly rinsed with physiological saline and follicular epithelium was dissected in physiological saline using the microsurgery technique. Next, the follicular epithelium was partially dissociated in 0.2% collagenase A (Sigma) for 15 min at 30°C. The isolated **e**pithelial cells were caught with a poly-L-lysine-coated cover slip, washed with physiological saline and incubated with 0.05 mM MDC for 15 min at room temperature in the dark. Determination of MDC-positive cells was performed immediately after preparation using a Nikon Eclipse TE 2000-U fluorescence microscope.

#### Acridine orange staining

For detection of lysosomes orange acridine staining was used. Ovaries from control- and peptide-injected females were stained with 1.6 μM acridine orange in saline for 5 min at room temperature in the dark. Next, ovaries were washed for 5 min in physiological saline, mounted using a mounting medium and examined with a Carl Zeiss LSM 510 confocal laser scanning microscope.

### Ultrastructural hallmarks of peptide-induced ovarian follicle degeneration

Ovaries from control- and peptide-injected females were dissected and fixed in 2.5% glutaraldehyde in 0.1 M phosphate buffer, pH 7.4 at room temperature for 2 h. They were rinsed in 0.1 M phosphate buffer and then the ovarioles were separated under a stereomicroscope. The ovarioles were post-fixed in 2% osmium tetroxide in the same buffer. The material was embedded in Epon 812 after dehydration in an ethanol and acetone series. Ultrathin sections were contrasted with uranyl acetate and lead citrate and examined under a JEM-1200 EX II transmission electron microscope.

## Competing interests

The authors declare that they have no competing interests.

## Authors’ contributions

EC designed the study, performed experiments, analyzed data and wrote the paper, GR designed the study and wrote the paper, EG performed EM experiments and analyzed the EM data, MK performed the synthesis of the peptide. All authors read and approved the final manuscript.

## Supplementary Material

Additional file 1 **Confocal micrographs of control (A) and the *Neb*-colloostatin-treated (B, C) terminal follicles of *T. molitor.*** Ovaries were stained with Oregon Green phalloidin for F-actin detection (green colour), in control oocyte F-actin is localized subcortically (arrow) and in follicular epithelium patency is visible **(A)**, in follicular epithelium of treated females *Neb*-colloostatin causes inhibition of intercellular space formation (arrow) **(B)**. Terminal follicle of treated females stained with SR-VAD-FMK shows caspase activity in oocyte **(C)**. Females were injected with saline (control) or 1 nmole *Neb*-colloostatin (treated) on day 3 and assayed on day 4. Scale bars: 20 μm. Click here for file
