# Supplementary figures and images for: The natural insect peptide Neb-colloostatin induces ovarian atresia and apoptosis in the mealworm Tenebrio molitor
Source: BMC Dev Biol. 2014 Jan 30;14:4. doi: 10.1186/1471-213X-14-4 (PMC3909444; doi:10.1186/1471-213X-14-4)

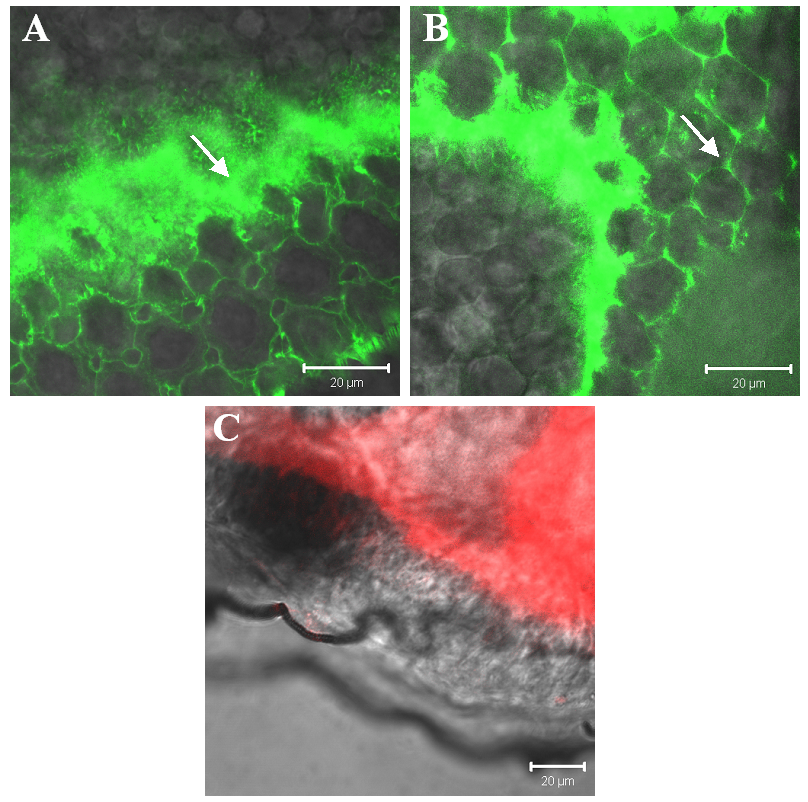

Supplement: Additional file 1 — Confocal micrographs of control (A) and the Neb -colloostatin-treated (B, C) terminal follicles of T. molitor. Ovaries were stained with Oregon Green phalloidin for F-actin detection (green colour), in control oocyte F-actin is localized subcortically (arrow) and in follicular epithelium patency is visible (A), in follicular epithelium of treated females Neb-colloostatin causes inhibition of intercellular space formation (arrow) (B). Terminal follicle of treated females stained with SR-VAD-FMK shows caspase activity in oocyte (C). Females were injected with saline (control) or 1 nmole Neb-colloostatin (treated) on day 3 and assayed on day 4. Scale bars: 20 μm. [file 1471-213X-14-4-S1.tiff]
